# Three-dimensional changes of scapulothoracic orientation in patients with acromioclavicular joint dislocations

**DOI:** 10.1016/j.jseint.2025.01.012

**Published:** 2025-02-11

**Authors:** Philipp Vetter, Sophie Frege, Alp Paksoy, Doruk Akgün, Markus Scheibel, Philipp Moroder

**Affiliations:** a Department of Trauma Surgery, University Hospital Zurich, Zurich, Switzerland; b BG Clinic, Trauma Hospital Berlin, Berlin, Germany; c Department of Shoulder and Elbow Surgery, Center for Musculoskeletal Surgery, Charité Universitaetsmedizin, Berlin, Germany; d Department of Shoulder and Elbow Surgery, Schulthess Clinic, Zurich, Switzerland

**Keywords:** Acromioclavicular, Scapula, Computed tomographic, Scapulothoracic, Three-dimensional, Dyskinesis, Rockwood

## Abstract

**Background:**

Acromioclavicular joint (ACJ) dislocations have been linked to altered scapulothoracic orientation and scapula dyskinesis, but research on three-dimensional (3D) changes in scapulothoracic orientation after such injury in vivo has not been described before. The aim of our pilot study was to analyze scapulothoracic orientation changes in patients with ACJ dislocations using three-dimensional computed tomographic (CT) image reconstruction.

**Methods:**

Patients with ACJ dislocations who underwent CT imaging were included retrospectively and consecutively. Minors and cases with spine, neurologic or systemic diseases, and shoulder girdle fractures were excluded. Each CT was performed in supine position with elbows rested on the scanning table and had to depict the complete shoulder girdle. After 3D image reconstruction, tilt, upward rotation, internal rotation, translation, and protraction of the scapula were measured based on three osseous landmarks: the glenoid (the deepest point of its concavity), the medial root of the scapular spine, and the inferior scapular angle. The healthy contralateral side was used as a paired control. ACJ dislocations were graded according to Rockwood (RW) on strict frontal CT image reconstruction, including the contralateral shoulder, where measurements were also performed to enable comparison. Cases were labeled as acute or chronic with a 3-week injury-to-diagnosis interval cut-off.

**Results:**

The mean age of the 14 patients (11 males and 3 females) was 38.6 ± 15.6 years (range, 18-71). Ten cases were defined as acute (RW types II: 1; III: 3; V: 6) and four as chronic (II: 1; III: 2; V: 1). On the injured side, the scapula showed more internal rotation (46.2° ± 5.3° vs. 42.1° ± 4.4°; *P* = .003), more scapular tilt (20.2° ± 4.6° vs. 17.9° ± 3.5°; *P* = .022), and less upward rotation (10.1° ± 3.6° vs. 12.0° ± 4.8°; *P* = .043). No difference between sides was found for scapular translation (*P* = .342) and scapular protraction (*P* = .385). There was a trend toward more internal rotation for RW type V injuries (*P* = .097).

**Conclusion:**

In this first 3D in vivo study, patients with ACJ dislocations displayed changes in scapulothoracic orientation in all planes. The scapula of the injured side was more internally rotated, forwardly tilted, and less upwardly rotated than on the healthy contralateral side.

Acromioclavicular joint (ACJ) dislocations are one of the most common injuries of the shoulder girdle.^18^

The ACJ connects the scapula to the clavicle and serves as a strut for brachial motion (especially overhead motion), relying on ligaments and muscle activation to provide stability and mobility^13^^,^^15^^,^^19^^,^^20^ in three axes simultaneously.^11^ After an ACJ dislocation, these musculoligamentous structures are disrupted, affecting joint stability and function^1^^,^^13^ to the point that the affected clavicular function can compromise scapular motion by impeding the kinematic chain.^2^

Scapular dyskinesis may occur due to several shoulder pathologies, including injury of the ACJ.^9^ It is defined as an abnormal orientation or scapulothoracic motion of the scapula^3^, ^4^, ^5^^,^^15^^,^^19^^,^^20^^,^^23^^,^^27^ that can manifest itself after several injuries to the shoulder area,^16^^,^^22^^,^^27^ often negatively affecting the clinical outcome.^4^^,^^7^ Additionally, it can provoke secondary symptoms^27^ such as pain and weakness.

The development of scapular dyskinesis has also been linked to ACJ dislocations,^7^ but research on three-dimensional (3D) changes in scapulothoracic orientation after such injury in vivo has not been described before. Identifying such changes could help to specifically address those in nonoperative or surgical treatment to restore anatomic orientation.

The aim of our study was to analyze the changes in scapulothoracic orientation in patients with ACJ dislocations using 3D computed tomographic (CT) image reconstruction.

## Materials and methods

In this retrospective case series, we searched the database of a university hospital (Charité – Universitätsmedizin Berlin, Berlin, Germany) for patients with ACJ dislocations displayed on conventional CT imaging between 2012 and 2020. The following inclusion criteria were required: (1) age 18 years or older, (2) patient in supine position during CT with elbows rested on the scanning table,^16^ (3) complete depiction of the shoulder girdle including full depiction of both shoulders with scapulae, indicating a lower vertebrae level of T7,^8^^,^^23^ (4) absence of spine pathologies, a history of systemic or neurological disease,^17^ or a previous shoulder history^23^ other than previous injury (treatment) on the affected ACJ.

The search yielded 17 cases. Three cases were excluded due to concomitant clavicle fractures (n = 2) or a coracoid fracture (n = 1), leaving 14 consecutive cases for analysis.

ACJ dislocations were graded according to Rockwood (RW)^25^ on CT imaging, including the contralateral shoulder, where measurements were also performed to enable comparison. The side-comparative coracoclavicular (CC) difference is 3 cm medial to the lateral clavicle^1^ of 10%-25% defined as RW type II, 25%-100% defined as RW type III, and >100% defined as RW type V. ACJ dislocations with an injury-to-diagnosis interval of a maximum of 3 weeks^13^ were defined as acute, while cases with an injury-to-diagnosis interval of more than 3 weeks were defined as chronic.

### Image reconstruction

After case anonymization, CT images of both scapulae were reconstructed in a 3D model using OsiriX Lite (Pixmeo SARL, Bernex, Switzerland). In a bone window level, surface rendering with complete subtraction of soft tissue and the scanning table was performed.^23^

CT modalities were identical in all patients, including a low- or full-dose imaging with a field of view, a tube voltage, and 120 kV (modulation ranging from 100 mA [low dose] or 200 mA [full dose] and a slice thickness of 0.625 mm or 1.25 mm [low and full dose] using a single scanner [Discovery MI; GE Healthcare, Chalfont St Giles, UK]).^16^^,^^23^

### Image measurements

For each of the 14 patients, a posteriorly viewed coronal, cranially viewed axillary and bilateral sagittal view with respect to the vertebral column were created for subsequent measurements on both scapulae, resulting in a total of 56 images for analysis.

**Figure 1:**
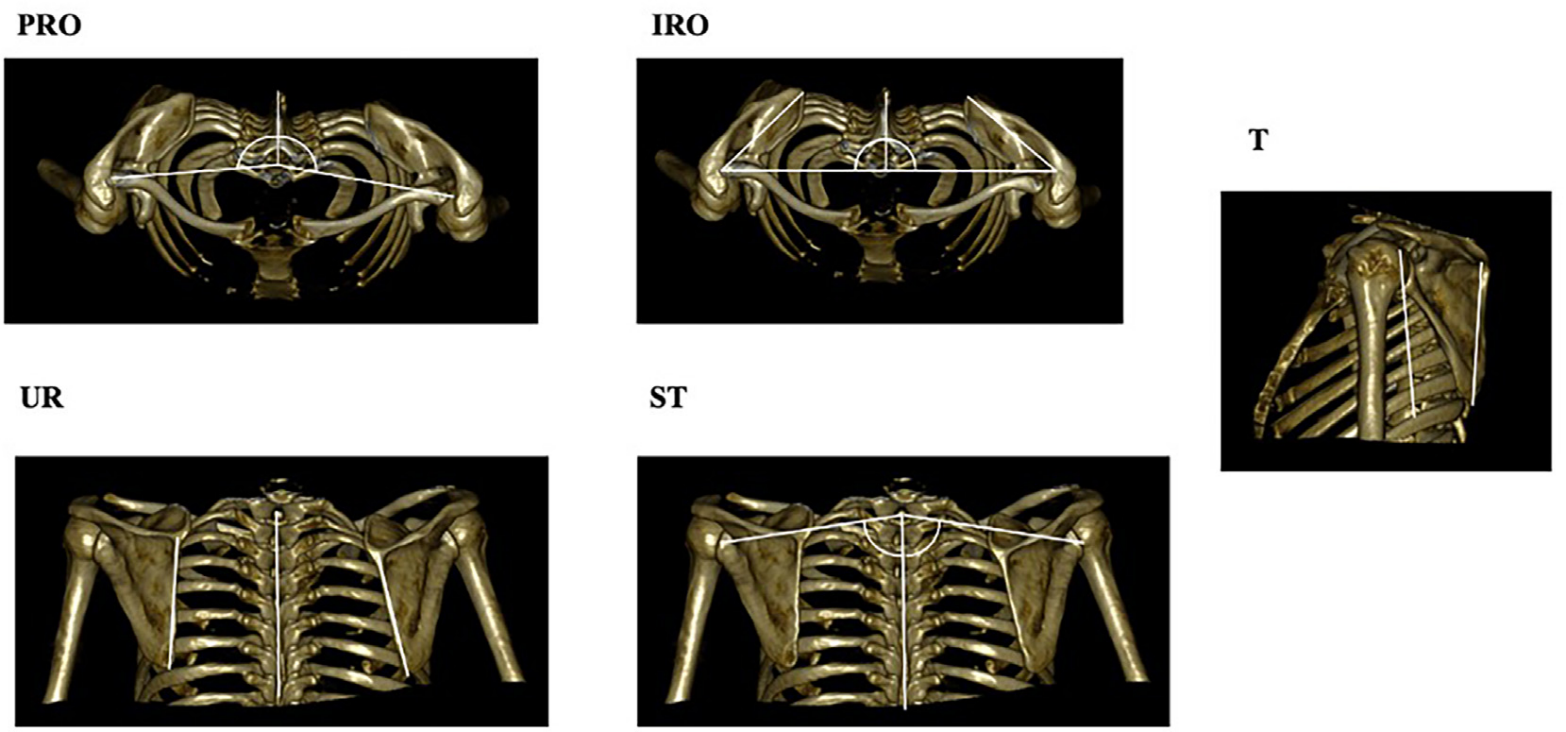
Three-dimensionally reconstructed images for measurement of scapular tilt (T), scapular upward rotation (UR), scapular translation (ST), scapular internal rotation (IR), and scapular protraction (PRO).

The parameters, as previously described by Moroder et al,^16^ included scapular tilt (T), scapular upward rotation (UR), scapular translation (ST), scapular internal rotation (IR), and scapular protraction (PRO) (Fig. 1). They were measured based on 3 osseous landmarks: the glenoid (the deepest point of its concavity), the medial root of the scapular spine, and the inferior scapular angle.^16^ The CT image acquisition included the contralateral shoulder as a paired control.

Bilateral sagittal views served for respective T measuring defined as the angle between the line running through the medial root of the scapular spine to the inferior scapular angle and the perpendicular line to the upper baseplate of vertebral body T1.

On posterior coronal views, UR was determined by measuring the angle between the vertebral column and a line crossing the medial root of the scapular spine and the inferior scapular angle.

ST was measured as the angle between the vertebral column and a line from vertebral body T1 to the glenoid.

On the axillary view from a superior perspective, IR was defined as the angle between the perpendicular line to the sagittal vertebral axis through vertebral body T1 to the glenoid and the medial root of the scapular spine. PRO was equal to the angle of a sagittal line through the vertebral body T1 and a line crossing the glenoid.

### Statistical analysis

Two observers (P.V. and S.F.) independently assessed all images at different time points (interobserver reliability). One observer (P.V.) repeated the measurements in anonymous and random order 4 weeks later (intraobserver reliability).

Statistical analysis was performed using SPSS 25.0 software (IBM Corp., Armonk, NY, USA). Intraclass correlation coefficient was calculated for each parameter in a two-way random manner with absolute agreement, being average measures (interobserver reliability) or single measures (intraobserver reliability). Grading of intraclass correlation coefficient was performed according to Landis and Koch^10^: A coefficient of 0.20 or less indicates slight agreement; 0.21-0.40, fair agreement; 0.41-0.60, moderate agreement; 0.61-0.80, substantial agreement; and 0.81 or greater, almost perfect agreement. For interrater reliability, the means of both raters were computed.

The Shapiro-Wilk test was used to test for normal distribution. Accordingly, the paired-t-test (normal distribution) or Wilcoxon-signed rank test (non-normal distribution) served for comparing the injured and healthy contralateral side. Comparisons between independent groups (according to chronicity [acute vs. chronic] or severity [RW types, comparing the combination of RW types II/III vs. type V^26^]) were performed using the Mann-Whitney U test. Values are presented as means with standard deviation, if not mentioned otherwise. The level of significance was set at *P* < .05.

All patients provided written consent for study inclusion. Ethical approval was granted by the local ethics committee (EA4/012/23).

## Results

In the cohort (11 males and 3 females), the mean age of the patients was 38.6 ± 15.6 years (range, 18-71).

There were 10 acute cases, comprising 1 case of RW type II, 3 cases of RW type III, and 6 cases of type V. Four cases were diagnosed as chronic, consisting of 1 type II, 2 type III, and 1 type V injury.

The CC distances of the injured and healthy sides were 16.2 ± 4.5 mm and 8.6 ± 2.2 mm, respectively (difference: 7.6 ± 3.9 mm). According to the RW classification, respective values for type II were 13.2 ± 3.0 mm and 10.8 ± 2.2 mm (2.4 ± 0.8 mm); for type III, 13.9 ± 4.0 mm and 8.4 ± 2.4 mm (5.5 ± 2.1 mm); and for type V, 18.8 ± 4.0 mm and 8.2 ± 1.9 mm (10.6 ± 2.5 mm).

Patient baseline characteristics are presented in Table I.

**Table I:** Patient baseline characteristics.

Patient number	Gender	Age	Injured side	Acute or chronic	RW type	Indication for CT	Concomitant injuries
1	Male	30	Left	A	II	Fall from height on arm (3 m)	Perilunate wrist luxation, radial head fracture
2	Male	27	Left	C	V	Bike accident	-
3	Male	32	Left	A	III	Fall after being tackled	-
4	Male	18	Right	A	V	Bike accident	Rib fractures, lung contusion
5	Male	40	Left	C	III	Previous arthroscopically assisted ACJ stabilization	-
6	Male	31	Right	A	III	Bike collision with car	Rib fractures
7	Male	31	Left	A	V	Bike accident	-
8	Female	68	Right	A	V	Fall with generalized pain	-
9	Male	46	Right	A	III	Bike accident	-
10	Male	40	Right	C	III	Previously failed Kirschner wire fixation of ACJ	-
11	Male	71	Right	A	V	Fall with generalized pain	Chest contusion
12	Female	28	Left	C	II	Previously failed hook plate	-
13	Female	51	Left	A	V	Hit by a car	Lower leg fracture
14	Male	27	Right	A	V	Fall from bike	Fracture of Os triquetrum

*A*, acute; *ACJ*, acromioclavicular; *C*, chronic; *CT*, computed tomography; *RW*, Rockwood.

Intraobserver reliability was almost perfect (Table II), while interobserver reliability was substantial to almost perfect (Table III).

**Table II:** Intraclass correlation coefficient with 95% confidence interval per measurement parameter for intraobserver reliability.

Measurement parameter	ICC	95% CI		Agreement
		Lower bound	Upper bound	
T	0.92	0.83	0.96	Almost perfect
UR	0.95	0.83	0.98	Almost perfect
ST	0.88	0.75	0.94	Almost perfect
IR	0.93	0.86	0.97	Almost perfect
PRO	0.94	0.87	0.97	Almost perfect

*CI*, confidence interval; *ICC*, intraclass correlation coefficient; *IR*, scapular internal rotation; *PRO*, scapular protraction; *ST*, scapular translation; *T*, scapular tilt; *UR*, scapular upward rotation.

**Table III:** Intraclass correlation coefficient with 95% confidence interval per measurement parameter for interobserver reliability.

Measurement parameter	ICC	95% CI		Agreement
		Lower bound	Upper bound	
T	0.88	0.75	0.95	Almost perfect
UR	0.92	0.82	0.96	Almost perfect
ST	0.79	0.55	0.90	Substantial
IR	0.89	0.67	0.96	Almost perfect
PRO	0.86	0.70	0.94	Almost perfect

*CI*, confidence interval; *ICC*, intraclass correlation coefficient; *IR*, scapular internal rotation; *PRO*, scapular protraction; *ST*, scapular translation; *T*, scapular tilt; *UR*, scapular upward rotation.

Comparing the healthy and injured sides, the scapula on the injured side showed more IR (*P* = .003) and T (*P* = .022) but less UR (*P* = .043) (Table IV). ST and PRO were not different (ST: *P* = .342; PRO: *P* = .385).

**Table IV:** Side-comparative parameters displayed as means with standard deviation (range), reported in degrees [°].

Parameter	Injured side	Healthy side	*P* value	Difference
T	20.2° ± 4.6° (15°-29°)	17.9° ± 3.5° (13°-24°)	**.022**	2.4° ± 3.0° (−2° to 6°)
UR	10.1° ± 3.6° (2°-18°)	12.0° ± 4.8° (3°-19°)	**.043**	−1.9° ± 3.4° (−8° to 5°)
IR	46.2° ± 5.3° (37°-54°)	42.1° ± 4.4° (34°-48°)	**.003**	4.1° ± 3.4° (−3° to 11°)
ST	78.6° ± 4.1° (71°-84°)	79.8° ± 3.7° (74°-85°)	.342	−1.1° ± 3.5° (−10° to 3°)
PRO	88.5° ± 4.0° (80°-93°)	89.1° ± 2.9° (82°-93°)	.385	0.6° ± 2.5° (−4° to 3°)

*IR*, scapular internal rotation; *PRO*, scapular protraction; *ST*, scapular translation; *T*, scapular tilt; *UR*, scapular upward rotation.

Further subgroup analysis was performed to compare acute and chronic cases as well as RW types II/III vs. type V injuries. Cases with an RW type V injury trended toward more IR (*P* = .097) (Tables V and VI).

**Table V:** Comparison of means of differences according to chronicity, displayed as means with standard deviation (range), reported in degrees [°].

Parameter	Acute subgroup	Chronic subgroup	*P* value
T	2.8° ± 3.1° (−2° to 6°)	1.2° ± 2.6° (−1° to 5°)	.374
UR	−1.3° ± 3.8° (−8° to 5°)	−3.3° ± 2.0° (5° to −1°)	.304
IR	5.1° ± 2.7° (2°-11°)	1.8° ± 4.0° (−3° to 6°)	.188
ST	−0.9° ± 4.1° (−10° to 3°)	−1.8° ± 1.9° (−3° to 1°)	.304
PRO	−0.4° ± 2.7° (−4° to 3°)	−1.3° ± 1.9° (−4° to 0°)	.839

*IR*, scapular internal rotation; *PRO*, scapular protraction; *ST*, scapular translation; *T*, scapular tilt; *UR*, scapular upward rotation.

**Table VI:** Comparison of means of differences according to injury severity, displayed as means with standard deviation (range), reported in degrees [°].

Parameter	Cases with RW type II/III injury	Cases with RW type V injury	*P* value
T	1.4° ± 1.9° (−1° to 5°)	3.3° ± 3.7° (−2° to 9°)	.259
UR	−1.3° ± 2.8° (−5° to 4°)	−2.4° ± 4.2° (−8° to 5°)	.535
IR	2.7° ± 2.9° (−3° to 6°)	5.6° ± 3.4° (0°-11°)	.097
ST	−0.4° ± 2.6° (−3° to 3°)	−1.9° ± 4.4° (−10° to 2°)	.710
PRO	−1.3° ± 2.4° (−4° to 3°)	0° ± 2.5° (−4° to 3°)	.383

*IR*, scapular internal rotation; *PRO*, scapular protraction; *RW*, Rockwood; *ST*, scapular translation; *T*, scapular tilt; *UR*, scapular upward rotation.

## Discussion

In this study, a 3D in vivo characterization of scapulothoracic orientation after ACJ dislocations was performed. The scapula on the injured side showed more IR and more T but less UR, without significant differences in PRO or ST. Cases with an RW type V injury trended toward more IR.

Since posture can affect scapulothoracic orientation^16^ and thereby the reference of measurements, the contralateral side served as control in this study. The values of the healthy side are accordant with previous reports.^15^^,^^19^^,^^24^

Side-comparative changes in T and UR are interpreted in the light of injuries to the ACJ ligament complex, predisposing to joint instability. In the intact state, the acromioclavicular and CC ligament complex provide a constraint to the anteroinferior tilt of the acromion.^12^^,^^19^^,^^20^^,^^24^ In cases of ligament injury due to ACJ dislocations, the constraint is at least partially lost and allows for more anterior (more T) and inferior (less UR) orientation, as described by Peeters et al in a seated cadaveric model resembling a Rockwood V lesion.^24^

In some cases, there seems to be a complete loss of the “bony bridge”^13^ between the lateral clavicle and the acromion with significant lowering^1^ and medial underriding^24^ of the acromion relative to the lateral clavicle. This missing abutment may then lead to a medial leaning of the scapula, as shown by more IR in our study. Peeters et al previously confirmed this interpretation by describing a more medial rotation of the scapula in the aforementioned seated cadaveric model.^24^

Cases with an RW type V injury trended toward more IR. The additional disruption of the deltotrapezial fascia likely aggravates the ACJ dissociation^1^^,^^13^^,^^21^ and thereby the measurable changes in this study.

Regarding the altered side-comparative parameters, side-comparative differences were generally more prominent for IR than for T and UR. This can also be seen as a result of the missing abutment of the acromion at the lateral clavicle, leading to a medial leaning of the scapula.^24^ The same mechanism can also explain that no changes in ST were found, as ST physiologically does not require such medial abutment.^24^

PRO is defined as the combination of IR, T, and ST.^9^ Despite the increase in IR and T, we see the following reasons for finding no side-comparative changes in PRO: patients were not advised to execute muscle activity, including no adduction movement, which was found to affect PRO.^24^ ST and PRO are mainly translation movements compared to the other parameters representing angulations.

Our findings support the assumption that ACJ dislocations are related to an altered scapulothoracic orientation. The better understanding of the direction of malrotation of the scapula after ACJ dislocation might help to further specify physiotherapeutic interventions and even surgical techniques to not only provide clavicle reduction but also improve the setting of the scapula.

Further studies employing 3D measurements of scapular motion are recommended, for instance, with magnetic resonance imaging or new motion analysis techniques optimized for scapulothoracic movement to identify scapular dyskinesis in relation to ACJ dislocations.

There are several limitations in our study. First, the case number is small. Second, the retrospective nature is associated with limited epidemiologic information (height, weight), and inclusion of patients with unusual imaging for this pathology may be associated with selection bias.

Third and foremost, the main limitation besides the small cohort is the supine patient positioning for CT-imaging, probably affecting scapulothoracic orientation due to gravity and mechanical barriers, combined with a static model coming short of a dynamic physiology. The biomechanical studies mentioned for comparison^19^^,^^20^ used cadavers positioned in a seated position compared to a flat supine position in our study. In an in vivo study investigating general changes of the shoulder girdle between the supine and standing position,^14^ patients in a standing position for CT imaging had less UR, T, and IR compared to measurements performed according to a supine position. In this regard, UR can generally exceed 30° in the scapulothoracic joint^6^ but appears limited in our model of a supine patient position. It is to be determined how scapulothoracic orientation in patients with ACJ dislocations is altered in an upright position, where arguably, the differences in scapulothoracic orientation observed in this trial are expected to be even greater. Any parameters should be referred to the healthy side for referencing.^16^^,^^26^

Fourth, soft-tissue status, including glenohumeral pathologies associated with altered scapulothoracic orientation, was not assessed. Concomitant injuries (especially rib fractures) or conditions altering vertical body axis like posture type^16^ and scoliosis may have affected scapulothoracic orientation. The extent of previous treatment (especially failed surgery in chronic cases) is unknown. In general, no causation can be inferred, and it is difficult to tell if the alterations in scapulothoracic orientation do provoke scapular dyskinesis.

As scapular dyskinesis has been shown to negatively affect the clinical outcome; however, the scapula setting should be in focus during nonoperative and even operative treatment of ACJ dislocations.

## Conclusion

In this first 3D in vivo study, patients with ACJ dislocations displayed changes in scapulothoracic orientation in all planes. The scapula of the injured side was more internally rotated, forwardly tilted, and less upwardly rotated than on the healthy contralateral side.

Future studies with a more rigorous level of evidence will still be needed before making a more definitive data-driven recommendation for endorsing 3D morphological assessments using CT scans to inform the clinical nonoperative and operative treatment decisions of ACJ dislocations.

## Disclaimers:

Funding: No funding was disclosed by the authors.

Conflicts of interest: Philipp Moroder is a consultant and receives royalties from Alyve Medical. All the other authors, their immediate families, and any research foundations with which they are affiliated have not received any financial payments or other benefits from any commercial entity related to the subject of this article.
